# Etymologia: Q Fever

**DOI:** 10.3201/eid1712.ET1712

**Published:** 2011-12

**Authors:** Nancy Männikkö

**Keywords:** Q fever, rickettsia, history, Edward Derrick, Coxiella burnetti, Australia

## Q fever [ku fe’vər]

 From Q for query, because the disease was an illness of unknown etiology. In 1937, Australian researcher Edward Derrick reported a disease that affected workers in slaughterhouses. *Coxiella burnetii*, the bacteria that causes the disease, was found shortly after Derrick’s investigation began. However, many aspects of the disease continue to puzzle researchers, making the name Q fever as appropriate today as it was 74 years ago.

Sources: Cooke RA. Q fever. Was Edward Derrick’s contribution undervalued? Med J Aust. 2008;189:660–2. PubMed; Derrick EH. “Q” fever, a new fever entity: clinical features, diagnosis, and laboratory investigation. Med J Aust. 1937;2:281–99; Dorland’s illustrated medical dictionary. 31st ed. Philadelphia: Saunders; 2007; Mackerras IM. Australian Academy of Sciences biographical memoirs of deceased members: Edward Holbrook Derrick 1898–1976 [cited 2011 Sep 9]. http://www.asap.unimelb.edu.au/bsparcs/aasmemoirs/derrick.htm#mac

